# Cdk5 Is Involved in BDNF-Stimulated Dendritic Growth in Hippocampal Neurons

**DOI:** 10.1371/journal.pbio.0050063

**Published:** 2007-03-06

**Authors:** Zelda H Cheung, Wing Hong Chin, Yu Chen, Yu Pong Ng, Nancy Y Ip

**Affiliations:** Department of Biochemistry, Biotechnology Research Institute and Molecular Neuroscience Center, Hong Kong University of Science and Technology, Clear Water Bay, Hong Kong, China; University of Cambridge, United Kingdom

## Abstract

Neurotrophins are key regulators of neuronal survival and differentiation during development. Activation of their cognate receptors, Trk receptors, a family of receptor tyrosine kinases (RTKs), is pivotal for mediating the downstream functions of neurotrophins. Recent studies reveal that cyclin-dependent kinase 5 (Cdk5), a serine/threonine kinase, may modulate RTK signaling through phosphorylation of the receptor. Given the abundant expression of both Cdk5 and Trk receptors in the nervous system, and their mutual involvement in the regulation of neuronal architecture and synaptic functions, it is of interest to investigate if Cdk5 may also modulate Trk signaling. In the current study, we report the identification of TrkB as a Cdk5 substrate. Cdk5 phosphorylates TrkB at Ser478 at the intracellular juxtamembrane region of TrkB. Interestingly, attenuation of Cdk5 activity or overexpression of a TrkB mutant lacking the Cdk5 phosphorylation site essentially abolishes brain-derived neurotrophic factor (BDNF)–triggered dendritic growth in primary hippocampal neurons. In addition, we found that Cdk5 is involved in BDNF-induced activation of Rho GTPase Cdc42, which is essential for BDNF-triggered dendritic growth. Our observations therefore reveal an unanticipated role of Cdk5 in TrkB-mediated regulation of dendritic growth through modulation of BDNF-induced Cdc42 activation.

## Author Summary

Accurate transmission of information in the nervous system requires the precise formation of contact points between neurons. Regulation of these contact sites involves fine tuning the number and branching of dendritic processes on neurons. Throughout development, several secreted factors act to regulate dendrite number and branching. One important family of these factors is neurotrophins, which are indispensable for the survival and development of neurons. For example, stimulation of hippocampal neurons with one neurotrophin, brain-derived neurotrophic factor (BDNF), increases the number of dendrites directly extending from the cell body. Here, we report that BDNF-stimulated dendritic growth requires phosphorylation of the BDNF receptor, TrkB, by a kinase known as cyclin-dependent kinase 5 (Cdk5). Inhibiting phosphorylation of TrkB by Cdk5 essentially abolishes the induction of dendrites by BDNF. Our observations reveal that Cdk5 serves as a regulator of neurotrophin function. Since Cdk5 and neurotrophins both play essential roles in neuronal development, our findings suggest that the interplay between Cdk5 and TrkB may also be implicated in the regulation of other biological processes during development.

## Abstract 3

Dendritic growth stimulated by brain-derived neurotrophic factor (BDNF) requires phosphorylation of the BDNF receptor, TrkB, by a kinase known as cyclin-dependent kinase 5 (Cdk5). This study identifies a novel interplay between Cdk5 and TrkB.

## Introduction

Neurotrophins are indispensable for multiple aspects of neuronal development, such as the maintenance of neuronal survival, regulation of neuronal architecture, and synaptic plasticity. Members of the neurotrophins include the prototypic member nerve growth factor (NGF), brain-derived neurotrophic factor (BDNF), neurotrophin (NT)–3, and NT-4/5. Downstream responses of neurotrophins are transduced by a family of receptor tyrosine kinases (RTKs) known as Trks, and also the low-affinity neurotrophin receptor p75. Although all neurotrophins bind p75, they associate with different Trk receptors with rather remarkable selectivity. NGF interacts selectively with TrkA, while BDNF and NT-4/5 bind preferentially to TrkB. NT-3, on the other hand, associates with TrkC with high affinity, although it also binds TrkA and TrkB with low affinity. Similar to other RTKs, activation of Trks leads to dimerization and autophosphorylation of the receptors, followed by the recruitment and initiation of a myriad of signaling pathways including the Ras/MAPK, PI3K, and PLCγ pathways [1,2].

Interestingly, recent studies have demonstrated that activity of cyclin-dependent kinase 5 (Cdk5), a serine/threonine kinase, is required for the downstream actions of a RTK, ErbB. Cdk5 was found to phosphorylate ErbB2/3, a phosphorylation that is essential for the activation of the receptors [3,4]. Cdk5 is a member of the cyclin-dependent kinase family, but it is unique in several aspects. First of all, it is activated by the neural-specific non-cyclin activators p35 and p39. Secondly, Cdk5 is not involved in the regulation of cell cycle control, but is implicated in neuronal migration, synapse functions/maintenance, and neuronal survival [5,6]. The importance of Cdk5 in neuronal development and migration is underscored by the aberrant phenotypes exhibited by mice lacking Cdk5 and its activators. Cdk5 knockout mice and p35/p39 double knockout mice both exhibit perinatal death with severe cortical lamination defects [7,8]. Furthermore, swollen soma and nuclear margination is evident in Cdk5-deficient neurons, implicating Cdk5 as an essential regulator of neuronal survival [7]. Interestingly, truncation of the Cdk5 activator p35 into p25 has also been associated with prolonged Cdk5 activation in a number of neurodegenerative diseases [9], thus revealing that precise regulation of Cdk5 activity is essential for maintenance of neuronal survival [10]. Furthermore, an increasing number of studies are pointing to an essential role of Cdk5 at the synapse, where it is not only involved in the formation and maintenance of synapses, but is also indispensable for the regulation of synaptic transmission and synaptic plasticity [5]. While the mechanisms by which Cdk5 regulates such diverse functions remain to be unraveled, the identification of ErbB receptors as Cdk5 substrates suggests that Cdk5 may exert its biological effects by modulating signaling pathways downstream of RTK activation. This piece of evidence, together with the abundant expression of Cdk5 and Trk receptors in the nervous system and their shared implication in a number of biological functions, prompted us to further examine if Cdk5 also regulates the signaling of Trk receptors.

In the current study, we report the identification of TrkB as a substrate of Cdk5. More importantly, we found that Cdk5-mediated phosphorylation of TrkB is essential for BDNF-induced dendritic growth through the modulation of Cdc42 activity. Our findings provide evidence for a crosstalk between the Cdk5 and neurotrophin signaling pathways, and lend further support to the idea that Cdk5 is a modulator of RTK signaling.

## Results

### TrkB Interacted with p35 and Cdk5

**Figure 1:**
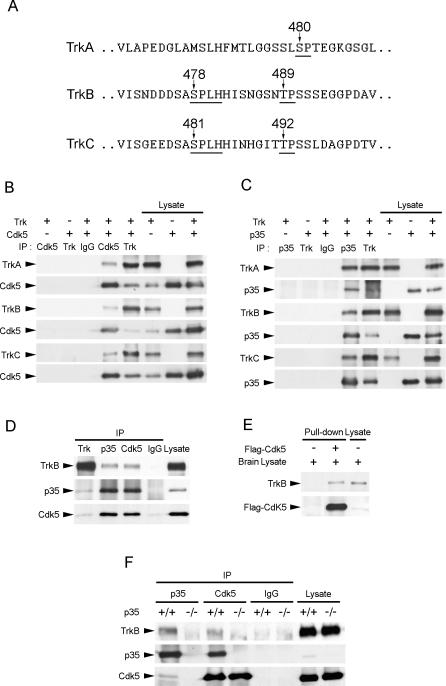
TrkB Interacted with Cdk5 and p35 (A) TrkA, TrkB, and TrkC all contain proline-directed serine/threonine residues in the juxtamembrane region of the receptors (indicated by arrows). Nonetheless, only TrkB and TrkC contain Cdk5 consensus sites S/TPXK/H/R. (B) Cell lysates from HEK293T cells overexpressing Cdk5 and TrkA, TrkB, or TrkC were immunoprecipitated (IP) with Cdk5 antibody and immunoblotted with pan-Trk antibody. TrkA, TrkB, and TrkC were all observed to associate with Cdk5. (C) Cell lysates from HEK293T cells overexpressing p35 and TrkA, TrkB, or TrkC were immunoprecipitated with p35 antibody and immunoblotted with pan-Trk antibody. TrkA, TrkB, and TrkC were all observed to associate with p35. (D) Brain lysate from P7 rat brain was immunoprecipitated with pan-Trk, p35, or Cdk5 antibody and immunoblotted with p35, Cdk5, and TrkB antibodies. Rabbit normal IgG was used as a control. TrkB was observed to associate with both p35 and Cdk5 in P7 rat brain. (E) The membrane fraction of adult brain lysates was incubated with or without Flag-tagged Cdk5. Flag-tagged Cdk5 pulled down TrkB from the membrane fraction of adult brain lysates. (F) Brain lysates from P7 p35^+/+^ or p35^−/−^ mouse brains were immunoprecipitated with p35 and Cdk5 antibodies and immunoblotted with p35, Cdk5, and TrkB antibodies. Rabbit normal IgG served as a control. Association between Cdk5 and TrkB was abolished in p35^−/−^ brain, indicating that p35 was required for the association between Cdk5 and TrkB.

Given the increasing evidence implicating Cdk5 in the modulation of RTK signaling, we sought to examine if Cdk5 may also play a role in Trk signaling. Literature search revealed that TrkA, TrkB, and TrkC all contain serine- or threonine-directed proline residues at the intracellular juxtamembrane region of the receptors, but only TrkB and TrkC contain Cdk5 consensus sites S/TPXK/H/R (Figure 1A). To explore the potential interplay between Trk receptors and Cdk5, we first examined if Trk receptors associated with Cdk5 or p35. TrkA, TrkB, or TrkC was overexpressed together with Cdk5 or p35 in COS7 cells, and immunoprecipitation was performed with Cdk5, p35, or pan-Trk antibody. Interestingly, all three Trk receptors were observed to associate with Cdk5 (Figure 1B) and p35 (Figure 1C), while no association was observed when immunoprecipitation was performed with IgG control. Since both TrkB and its ligand BDNF are abundantly expressed in the brain throughout development, we next proceeded to verify the interaction between TrkB and Cdk5/p35 in postnatal brains. We found that TrkB associated with both p35 and Cdk5 in postnatal day 7 (P7) rat brain lysates (Figure 1D). Furthermore, Flag-tagged Cdk5 pulled down TrkB from the membrane fraction of adult brain lysates (Figure 1E). These observations collectively suggest that TrkB interacted with Cdk5/p35 in both postnatal and adult brains. Since both p35 and Cdk5 are present in brain lysates and likely exist as a complex, the observed interaction between TrkB and Cdk5/p35 did not provide specific information on whether TrkB associated specifically with Cdk5 or p35. To delineate between these two possibilities, the interaction between TrkB, p35, and Cdk5 was examined in p35^+/+^ and p35^−/−^ brain lysates (Figure 1F). Interestingly, we found that in the absence of p35, the association between Cdk5 and TrkB was essentially abolished, indicating that p35 was required for the association between Cdk5 and TrkB in vivo.

### Cdk5 Phosphorylated TrkB at Ser478

**Figure 2:**
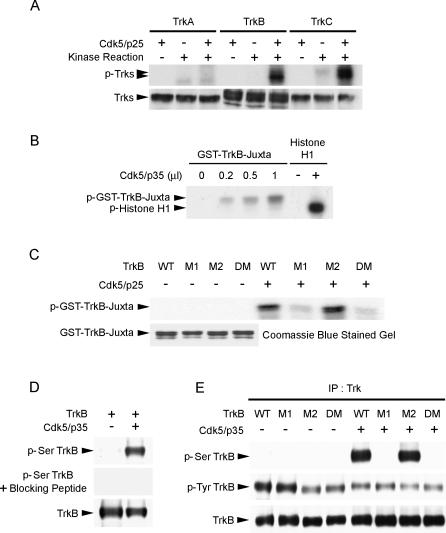
Cdk5 Phosphorylated TrkB at Ser478 (A) Lysates from COS7 cells overexpressing TrkA, TrkB, and TrkC were immunoprecipitated with pan-Trk antibody and incubated with Cdk5/p25 in an in vitro kinase assay. TrkB and TrkC, but not TrkA, were phosphorylated by Cdk5/p25. (B) GST-TrkB-juxtamembrane fusion protein was incubated with increasing amount of Cdk5/p35 and subjected to an in vitro kinase assay. Histone H1 served as control to verify the activity of the Cdk5 kinase. The TrkB-juxtamembrane region was phosphorylated by Cdk5/p35 in a dose-dependent manner. (C) Purified WT GST-TrkB-juxtamembrane fusion protein and mutants (M1, M2, and DM) were incubated with Cdk5/p25 in an in vitro kinase assay. While WT and M2 were strongly phosphorylated by Cdk5/p25, phosphorylation of M1 and DM were markedly attenuated. Quality of the purified GST and GST-fusion proteins used in the GST pull-down assay was verified by Coomassie blue staining. (D) Characterization of p-Ser TrkB antibody raised against phosphorylated Ser478 of TrkB. TrkB was overexpressed with or without p35/Cdk5 in HEK293T cells. Preincubation of purified p-Ser478 TrkB antibody with blocking peptide completely abolished detection of Ser478 phosphorylation of TrkB. (E) Full-length TrkB WT, M1, M2, and DM were overexpressed with or without Cdk5/p35 in HEK293T cells. In the absence of Cdk5/p35, Ser478-phosphorylated TrkB (p-Ser TrkB) was not detected. Overexpression of Cdk5/p35 resulted in phosphorylation of TrkB WT at Ser478, but phosphorylation at Ser478 was essentially abolished when TrkB M1 and DM were overexpressed. IP, immunoprecipitation.

We next proceeded to examine if Trk receptors, TrkB in particular, served as Cdk5 substrates using in vitro kinase assay. TrkA, TrkB, and TrkC were overexpressed in COS7 cells and immunoprecipitated by pan-Trk antibody. Incubation with Cdk5/p25 revealed that TrkB and TrkC, but not TrkA, were phosphorylated by Cdk5/p25 in vitro (Figure 2A). This is in agreement with the lack of Cdk5 consensus sites in TrkA, and points to the possibility that Cdk5 may phosphorylate TrkB and TrkC at the Cdk5 consensus sites at the juxtamembrane region (Figure 1A). To examine this possibility, a GST fusion protein containing only the juxtamembrane region of TrkB was prepared. In vitro kinase assay verified that Cdk5/p35 phosphorylated TrkB at the juxtamembrane region (Figure 2B). It has previously been proposed that p25 and p35 may confer different substrate specificities. Results from our in vitro kinase assay suggested that Cdk5 phosphorylated TrkB regardless of whether it was activated by p25 or p35, although further studies will be required to delineate the relative contributions of p25 and p35 to endogenous phosphorylation of TrkB by Cdk5.

We were next interested in identifying the Cdk5 phosphorylation site(s) on TrkB. Three TrkB-juxtamembrane region mutants were generated: TrkB M1, where Ser478 was mutated to alanine; TrkB M2, where Thr489 was mutated to alanine; and TrkB DM, where both Ser478 and Thr489 were mutated to alanine. Interestingly, phosphorylation of the TrkB-juxtamembrane region was almost completely abolished when Cdk5/p25 was incubated with TrkB M1 or TrkB DM (Figure 2C), thus revealing that Ser478 was required for Cdk5-mediated phosphorylation of the TrkB-juxtamembrane region. We further verified the importance of this site for Cdk5-mediated phosphorylation of TrkB by generating a phospho-specific TrkB antibody against Ser478. Preincubation of the antibody with blocking peptide prevented detection of Ser478-phosphorylated TrkB, indicating that the antibody was sufficiently specific (Figure 2D). Full-length TrkB mutants lacking the potential Cdk5 phosphorylation sites were overexpressed with or without Cdk5/p35 in HEK293T cells. Interestingly, Ser478-phosphorylated TrkB was not observed in the absence of Cdk5/p35, indicating that Cdk5 was essential for the phosphorylation of TrkB at Ser478 in HEK293T cells. More importantly, when TrkB mutants lacking Ser478 were expressed (TrkB M1 and TrkB DM), phosphorylation of TrkB at Ser478 was essentially abolished (Figure 2E). Taken together, our observations indicate that Cdk5 phosphorylated TrkB at Ser478 at the juxtamembrane region of TrkB.

### Ser478 Phosphorylation of TrkB Required Cdk5 Activity In Vivo

**Figure 3:**
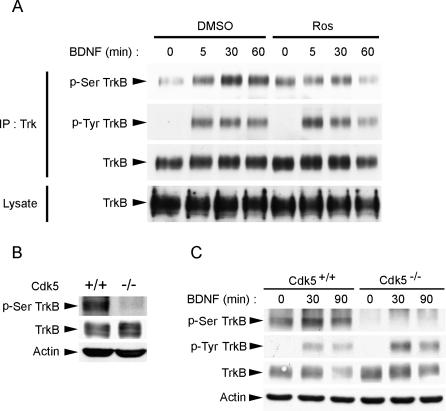
Ser478 Phosphorylation of TrkB Required Cdk5 Activity In Vivo (A) BDNF stimulation resulted in an increase in p-Ser478 TrkB (p-Ser TrkB) levels in cortical neurons. Treatment with Cdk5 selective inhibitor Ros (25 μM) inhibited the BDNF-induced increase in p-Ser478 TrkB, although Ros treatment also resulted in a slight increase in basal p-Ser478 TrkB. (B) *cdk5* ^+/+^ and *cdk5* ^−/−^ brain lysates were immunoblotted against TrkB, phospho-TrkB at Ser478, and β-actin as loading control. p-Ser478 TrkB was almost completely absent in *cdk5* ^−/−^ brain, indicating the importance of Cdk5 in the phosphorylation of TrkB at Ser478 in vivo. (C) Cortical neurons isolated from *cdk5* ^+/+^ and *cdk5* ^−/−^ brain were treated with BDNF for different periods. Interestingly, while BDNF enhanced TrkB Ser478 phosphorylation in *cdk5* ^+/+^ cortical neurons, TrkB Ser478 phosphorylation was not detected in *cdk5* ^−/−^ neurons, nor did BDNF stimulation enhance Ser478 phosphorylation, indicating that BDNF-stimulated increase in TrkB Ser478 phosphorylation requires Cdk5 activity.

To further examine if Cdk5 is essential for phosphorylation of TrkB at Ser478 in vivo, we examined the effect of inhibiting Cdk5 activity on phospho-Ser478 (p-Ser478) TrkB levels in cortical neurons. We found that at basal level, TrkB was weakly phosphorylated at Ser478. Interestingly, stimulation with BDNF led to a marked increase in p-Ser478 TrkB levels, indicating that phosphorylation of TrkB at Ser478 was at least in part ligand dependent. Remarkably, treatment with Cdk5 selective inhibitor roscovitine (Ros) almost abrogated the BDNF-triggered increase in p-Ser478 TrkB levels (Figure 3A), suggesting that Cdk5 was involved in the BDNF-stimulated component of TrkB Ser478 phosphorylation. To further establish the involvement of Cdk5 in Ser478 phosphorylation of TrkB in vivo, the levels of p-Ser478 TrkB in *cdk5* ^+/+^ and *cdk5* ^−/−^ brain lysates were examined. Importantly, we found that Ser478-phosphorylated TrkB was basically undetectable in Cdk5^−/−^ brain lysates (Figure 3B). Similarly, cortical neurons prepared from Cdk5^−/−^ brains exhibited undetectable levels of p-Ser478 TrkB. In addition, BDNF stimulation failed to trigger an increase in p-Ser478 TrkB levels (Figure 3C). These observations strongly suggest that Cdk5 is essential for phosphorylation of TrkB at Ser478 in vivo, and that BDNF-stimulated increase in Ser478 phosphorylation of TrkB requires Cdk5 activity.

### BDNF Treatment Enhanced Cdk5 Activity

**Figure 4:**
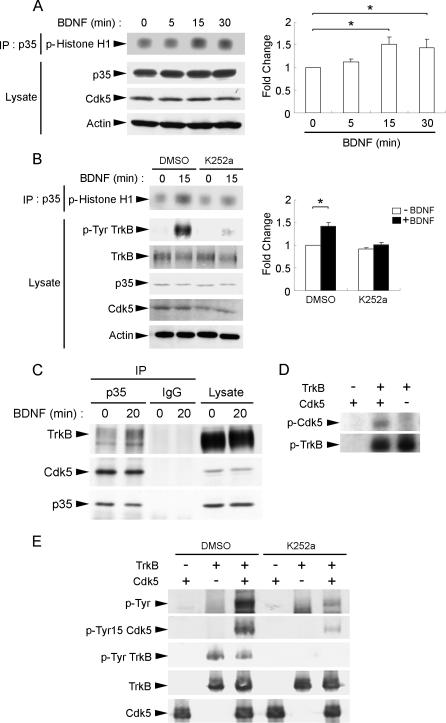
BDNF Enhanced Cdk5 Activity (A) Cortical neurons were stimulated with BDNF for different time intervals. Lysates were immunoprecipitated (IP) with p35 antibody and subjected to in vitro kinase assay using histone H1 as substrate. BDNF stimulation for 15 min resulted in a marked increase in Cdk5 activity in cortical neurons. Quantification of the changes in phospho-Histone H1 level following BDNF stimulation was normalized to the value obtained from untreated cultures (time 0) and is shown in the histogram. *, *p <* 0.05. (B) Addition of Trk inhibitor K252a abolished BDNF-induced increase in Cdk5 activity. Cortical neurons were pretreated with vehicle control (DMSO) or K252a for 30 min before stimulation with BDNF for 15 min. Lysates were immunoprecipitated with p35 antibody and subjected to in vitro kinase assay using histone H1 as substrate. We found that K252a pretreatment markedly reduced the increase in Cdk5 activity triggered by BDNF stimulation, indicating that the induction of Cdk5 activity was dependent on TrkB activation. Quantification of the changes in phospho-Histone H1 level following BDNF stimulation in the presence or absence of K252a treatment was normalized to the value obtained from untreated cultures (time 0) and is shown in the histogram. *, *p <* 0.05. (C) Cortical neurons were treated with BDNF for 20 min. Lysates were immunoprecipitated with p35 antibody and immunoblotted with TrkB, p35, or Cdk5 antibody. While association between Cdk5 and p35 was not affected by BDNF stimulation, association between p35 and TrkB increased following 20 min of BDNF stimulation. (D) Recombinant TrkB was incubated with GST-Cdk5 in an in vitro kinase assay. TrkB was found to phosphorylate GST-Cdk5 (middle lane). (E) GST-Cdk5 and recombinant TrkB were pretreated with vehicle (DMSO) or K252a for 10 min, subjected to in vitro kinase assay, and immunoblotted with antibodies against phospho-tyrosine (p-Tyr) and the Tyr15 phosphorylated form of Cdk5 (pTyr15 Cdk5). Cdk5 was phosphorylated by TrkB at Tyr15. Addition of K252a abolished phosphorylation of Cdk5 by TrkB, further verifying that Cdk5 was phosphorylated by TrkB.

Since BDNF stimulation was observed to increase Ser478 phosphorylation of TrkB, and Cdk5 was required for phosphorylating TrkB at Ser478, we were interested to examine if BDNF stimulation affects Cdk5 activity. BDNF has previously been observed to increase Cdk5 activity after 3 d of BDNF stimulation in cortical neurons [11]. In agreement with this observation, we found that BDNF treatment led to an increase in Cdk5 activity within 15 min of BDNF stimulation (Figure 4A). More importantly, addition of Trk inhibitor K252a essentially abolished BDNF-triggered increase in Cdk5 activity, indicating that the increase in Cdk5 activity was dependent on TrkB activation (Figure 4B). It has previously been demonstrated that Cdk5 activity is enhanced by phosphorylation at Tyr15 [12]. Given the activation of tyrosine kinase activity of TrkB upon ligand stimulation, we were interested to investigate if BDNF treatment leads to phosphorylation of Cdk5 at Tyr15, thereby enhancing its activity. We found that BDNF stimulation enhanced association between Cdk5 and TrkB in cortical neurons (Figure 4C). More importantly, in vitro kinase assay using purified TrkB and Cdk5 revealed that TrkB phosphorylated Cdk5 at Tyr15 (Figure 4D and 4E). TrkB-mediated phosphorylation of Cdk5 was abolished with the addition of Trk inhibitor K252a, further verifying that Tyr15 phosphorylation of Cdk5 was TrkB dependent (Figure 4E). These observations collectively indicate that upon BDNF stimulation, Cdk5 was recruited to TrkB and phosphorylated by TrkB at Tyr15, thus leading to enhanced Cdk5 activity to promote phosphorylation of TrkB at Ser478.

### Ser478 Phosphorylation of TrkB Was Required for BDNF-Stimulated Dendritic Growth

Given the neural-specific nature of Cdk5, its abundant expression throughout development, and its essential role in the phosphorylation of TrkB at Ser478, we were interested in examining the biological significance of this phosphorylation on the downstream functions of BDNF/TrkB signaling. As a first step, we examined if Cdk5-mediated phosphorylation of TrkB affects TrkB activation and downstream signaling cascades. Interestingly, we found that inhibition of Cdk5 activity by Cdk5 selective inhibitor Ros only marginally affected tyrosine phosphorylation of TrkB and initiation of downstream signaling pathways including phosphorylation of Erk1/2, Akt, and CREB (data not shown). Indeed, BDNF-stimulated increase in TrkB tyrosine phosphorylation was weakly affected in *cdk5* ^−/−^ cortical neurons (Figure 3C). Furthermore, activation of Akt and Erk1/2 following BDNF stimulation was also comparable in *cdk5* ^+/+^ and *cdk5* ^−/−^ cortical neurons (data not shown). Our observations thus revealed that Cdk5-mediated phosphorylation of TrkB did not significantly affect activation of the receptor, nor its initiation and recruitment of downstream signaling pathways.

**Figure 5:**
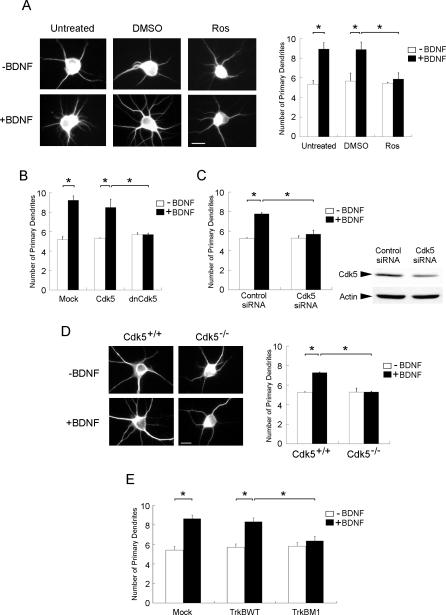
Attenuation of Cdk5 Activity Abolished BDNF-Induced Increase in Primary Dendrites in Hippocampal Neurons (A) Hippocampal neurons were stimulated with BDNF for 3 d in the presence or absence of Ros (10 μM). Interestingly, while BDNF treatment markedly enhanced the number of primary dendrites, treatment with Ros abrogated the increase. (B) Hippocampal neurons were transfected with Cdk5 or DN Cdk5. Twenty-four hours after transfection, cells were exposed to BDNF for 3 d. Overexpression of DN Cdk5 abolished the BDNF-induced increase in primary dendrites. (C) Hippocampal neurons were transfected with Cdk5 siRNA or control siRNA. Twenty-four hours after transfection, cells were exposed to BDNF for 3 d. Transfection with Cdk5 siRNA attenuated Cdk5 expression in hippocampal neurons. More importantly, BDNF-induced increase in primary dendrites was abrogated in Cdk5 siRNA–transfected cells. (D) Hippocampal neurons isolated from *cdk5* ^+/+^ and *cdk5* ^−/−^ brains were treated with BDNF for 3 d. BDNF treatment failed to enhance primary dendrites in Cdk5^−/−^ neurons. (E) Hippocampal neurons were transfected with TrkB WT or TrkB M1. Twenty-four hours after transfection, cells were exposed to BDNF for 3 d. Overexpression of TrkB M1 markedly reduced the BDNF-induced increase in primary dendrites. Scale bar = 10 μm. *, *p <* 0.05.

Although Cdk5-mediated phosphorylation of TrkB had negligible effect on the downstream signaling of TrkB, it cannot be ruled out that Ser478 phosphorylation of TrkB is essential for the downstream functions of BDNF/TrkB signaling. We thus sought to examine if Cdk5-mediated phosphorylation of TrkB affects its downstream functions. BDNF has been observed to stimulate dendrite growth and development in hippocampal neurons [13,14]. In accordance with earlier observations, BDNF treatment led to a marked increase in the number of primary dendrites in hippocampal neurons (Figure 5A), although the length and branching of dendrites were not affected (data not shown). Interestingly, treatment with Cdk5 selective inhibitor Ros almost completely abolished the BDNF-stimulated dendritic growth, without affecting the basal number of dendrites (Figure 5A). Furthermore, overexpression of dominant negative (DN) Cdk5 (Figure 5B) and transfection with Cdk5 short interfering RNA (siRNA) (Figure 5C) both abrogated BDNF-induced increase in primary dendrites. More importantly, BDNF similarly failed to induce an increase in primary dendrites in *cdk5* ^−/−^ hippocampal neurons (Figure 5D). These observations collectively reveal that Cdk5 activity was required for BDNF-induced increase in primary dendrites in hippocampal neurons. To verify the importance of Ser478 phosphorylation of TrkB in BDNF-triggered dendritic growth, TrkB wild-type (WT) or TrkB M1 was overexpressed in hippocampal neurons. Remarkably, overexpression of TrkB M1 similarly abolished the BDNF-induced increase in primary dendrites (Figure 5E). Taken together, our data indicate that Cdk5-mediated phosphorylation of TrkB at Ser478 was required for BDNF-triggered dendritic growth in hippocampal neurons.

### Cdk5-Mediated Phosphorylation of TrkB Affected BDNF-Induced Dendritic Growth through Attenuation of Cdc42 Activity

**Figure 6:**
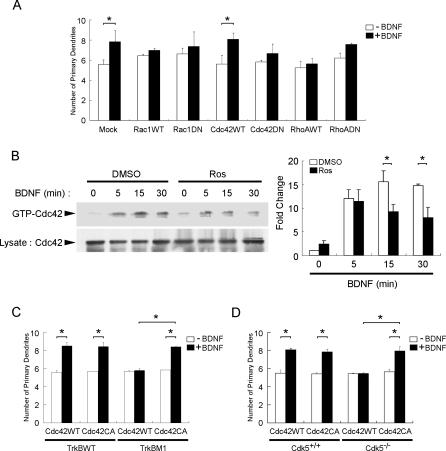
Cdk5-Mediated Phosphorylation of TrkB Affected BDNF-Induced Dendritic Growth through Attenuation of Cdc42 Activity (A) Hippocampal neurons were transfected with the WT or DN form of Rac1, RhoA, or Cdc42. Twenty-four hours after transfection, cells were exposed to BDNF for 3 d. Overexpression of DN Cdc42 markedly reduced BDNF-induced increase in primary dendrites compared to overexpression of WT Cdc42, indicating that Cdc42 may contribute to the BDNF-dependent induction of dendritic growth in hippocampal neurons. (B) Cortical neurons were pretreated with Cdk5 selective inhibitor Ros or vehicle (DMSO) for 30 min prior to treatment with BDNF for different time intervals. Ros pretreatment markedly reduced BDNF-induced increase in Cdc42 activity following 15 and 30 min of BDNF treatment, indicating that Cdk5 activity was involved in BDNF-triggered Cdc42 activation. Quantification of the changes in Cdc42 activity following BDNF stimulation with or without Ros pretreatment was normalized to the value obtained for the DMSO-treated group at time 0 and is shown in the histogram. *, *p <* 0.05. (C) TrkB WT or TrkB M1 mutant were co-transfected with WT or CA Cdc42 in hippocampal neurons. Twenty-four hours after transfection, cells were exposed to BDNF for 3 d. Overexpression of CA Cdc42 reversed the abrogation of BDNF-induced increase in primary dendrites following overexpression of TrkB M1. *, *p <* 0.05. (D) Hippocampal neurons isolated from *cdk5* ^+/+^ and *cdk5* ^−/−^ brains were transfected with WT or CA Cdc42. Twenty-four hours after transfection, cells were exposed to BDNF for 3 d. Overexpression of CA Cdc42 rescued the lack of dendritic growth following BDNF treatment in Cdk5^−/−^ hippocampal neurons. *, *p <* 0.05.

Rho GTPases, including RhoA, Rac1, and Cdc42, are key regulators of actin cytoskeleton dynamics. Since BDNF stimulation has been observed to activate Rac1 and Cdc42 in neurons [15], we were interested to delineate if Rho GTPases contribute to BDNF-stimulated dendritic growth. To investigate if Rho GTPases are involved, and to identify the Rho GTPase(s) implicated, hippocampal neurons were transfected with WT or DN Rac1, Cdc42, or RhoA. We found that while overexpression of WT and DN Rac1 increased the basal number of dendrites in the absence of BDNF treatment, overexpression of both forms of Rac1 abolished BDNF-stimulated dendritic growth. On the other hand, while overexpression of DN RhoA slightly enhanced primary dendrites irrespective of BDNF stimulation, overexpression of both WT and DN forms of RhoA inhibited BDNF-stimulated dendritic growth. Remarkably, in contrast to the inhibition of BDNF-stimulated dendritic growth in cells overexpressing WT Rac1 and RhoA, BDNF stimulation of hippocampal neurons overexpressing WT Cdc42 resulted in an increase in primary dendrites, which was nearly abolished by overexpression of DN Cdc42 (Figure 6A). Our observations therefore suggest that while Rac1 and RhoA may also modulate BDNF-stimulated dendritic growth, it is the activation of Cdc42 following BDNF stimulation that most likely mediates the increase in primary dendrites by BDNF.

To examine if phosphorylation of TrkB by Cdk5 affects dendritic growth through modulating BDNF-triggered activation of Cdc42, we first examined if the BDNF-induced increase in Cdc42 activity was affected by treatment with Cdk5 selective inhibitor Ros. In agreement with earlier findings, BDNF treatment resulted in an increase in Cdc42 activity. Interestingly, treatment with Ros significantly reduced BDNF-induced Cdc42 activity in cortical neurons (Figure 6B), suggesting that Cdk5 activity was involved in BDNF-triggered activation of Cdc42. To investigate if the reduction in Cdc42 activity contributes to the abrogation of BDNF-induced dendritic growth following attenuation of Cdk5 activity, the effect of overexpressing constitutively active (CA) Cdc42 with TrkB M1 on BDNF-induced dendritic growth was examined. Remarkably, overexpression of CA Cdc42 reversed the abrogation of BDNF-induced dendritic growth by TrkB M1 (Figure 6C). More importantly, while overexpression of CA Cdc42 had negligible effect on BDNF-stimulated increase in primary dendrites in Cdk5^+/+^ neurons, overexpression of CA Cdc42 similarly rescued the lack of dendritic growth in *cdk5* ^−/−^ neurons following BDNF stimulation (Figure 6D). These observations strongly suggest that Cdk5-mediated phosphorylation of TrkB at Ser478 was essential for the BDNF-triggered increase in primary dendrites through modulating BDNF-induced Cdc42 activity.

## Discussion

In the current study, we report the identification of TrkB as a novel Cdk5 substrate by providing evidence that Cdk5 phosphorylates TrkB at Ser478, located at the intracellular juxtamembrane region of the receptor. The near absence of Ser478-phosphorylated TrkB in *cdk5* ^−/−^ brain underscores the importance of Cdk5 in this phosphorylation in vivo. More importantly, we found that Cdk5-mediated phosphorylation of TrkB is required for BDNF-stimulated increase in primary dendrites. Furthermore, we demonstrated that Cdk5 activity is involved in BDNF-induced increase in Cdc42 activity, which underlies BDNF-induced dendritic growth in hippocampal neurons. Overexpression of CA Cdc42 restored BDNF-stimulated increase in primary dendrites in *cdk5* ^−/−^ neurons, lending further support that Cdk5-mediated phosphorylation of TrkB at Ser478 is essential for BDNF-induced Cdc42 activation and increase in primary dendrites. Our findings therefore reveal an unanticipated role of Cdk5 in mediating downstream functions of Trk signaling.

Activation of Rho GTPases has been implicated in a number of functions downstream of neurotrophin stimulation. For example, a recent study reported that synaptic maturation involves BDNF-stimulated increase in Cdc42 activity [16]. In addition, activation of Cdc42 is involved in the regulation of retinal growth cone filopodia by BDNF [17]. Activation of Rac1 following neurotrophin stimulation has also been observed to mediate neuronal migration triggered by neurotrophin treatment [18]. Our observation that BDNF-stimulated increase in Cdc42 activity contributes to the increase in primary dendrites corroborates these studies. It is interesting to note that overexpression of WT and DN Rac1 and RhoA also inhibited BDNF-induced increase in primary dendrites. While it is rather intriguing to observe similar actions by the WT and DN forms of these two Rho GTPases, our observation nonetheless suggests that Rac1 and RhoA may also play a role in BDNF-stimulated dendritic growth. Further studies will be required to delineate their involvements in BDNF-dependent regulation of dendritic development.

Although different Rho GTPases have been identified as essential downstream mediators of neurotrophin functions, much less is known about the mechanisms by which neurotrophin treatment results in Rho GTPase activation, and how this process is regulated. The activity of Rho GTPases is controlled by a number of factors. Conversion from the GDP-bound, inactive state to the GTP-bound, active state is facilitated by guanine nucleotide exchange factors (GEFs). The activated Rho GTPases then translocate to the plasma membrane, where they activate other downstream effectors such as PAK1 to modulate actin dynamics [19]. Indeed, neurotrophins have recently been observed to induce Rho GTPase activity through recruitment of a number of GEFs. TrkA was demonstrated to bind to Kalirin, an association that is essential for NGF-induced Rac1 activation and neurite outgrowth [20]. Furthermore, NGF treatment induces plasma membrane translocation of the GEFs Vav2 and Vav3, an event that is required for activation of Rac1 and Cdc42 and the induction of neurite outgrowth following NGF treatment in PC12 cells [21]. NGF also stimulates activation of the Rac-specific GEF p-Rex1 in PC12 cells [18]. Two recent studies reveal that neurotrophin stimulation in Schwann cells also leads to Rho GTPase activation through activation of GEFs. TrkC activation results in activation of the Cdc42-specific GEF Dbs [22] and Rac-specific GEF Tiam1 [23], both of which are required for NT-3-stimulated Schwann cell migration. Finally, TrkB was also recently demonstrated to bind and phosphorylate Tiam1 to mediate a BDNF-triggered change in cell shape [24].

On the other hand, recent studies accentuate the importance of membrane recruitment of Rho GTPase to lipid rafts for the function of these Rho GTPases. Lipid rafts are microdomains in plasma membrane rich in cholesterol and sphingolipids. Targeting of activated Rac1 to lipid rafts is required for activation of downstream effector Pak1 [25]. More importantly, neurotrophin-triggered Rac1 activation and morphological changes in hippocampal neurons have also been observed to require localization of Rac1 to lipid rafts [26]. Finally, BDNF has also been observed to increase Cdc42 activity in cerebellar granule neurons through enhancing calcium influx following the activation of PLCγ and PI3K pathways, a series of events that are essential for BDNF-mediated growth cone turning [27]. While a number of mechanisms have been postulated to underlie neurotrophin-mediated activation of Rho GTPases, it appears that the mechanisms implicated may vary with different downstream functions of Trk activation and the GEF involved.

In the current study, we demonstrated that Ser478 phosphorylation of TrkB by Cdk5 is essential for the Cdc42-dependent increase in primary dendrites triggered by BDNF, thus adding a new regulatory component to the mechanisms involved in Rho GTPase activation by neurotrophin. Although the precise downstream pathways by which this phosphorylation affects Cdc42 activation remains to be determined, our observations provide some interesting insights. First of all, while inhibition of Cdk5-mediated TrkB phosphorylation at Ser478 essentially abolished BDNF-induced increase in primary dendrites, it was surprising to observe that Cdk5 activity had a negligible effect on TrkB activation and initiation of downstream signaling pathways. This suggests that Cdk5 activity probably did not affect BDNF-dependent activation of Cdc42 and the induction of primary dendrites through modulating activation of downstream signaling. This is unexpected because BDNF-stimulated increase in primary dendrites was previously observed to depend on PI3K/Akt pathways in cortical neurons [28]. Nonetheless, accumulating evidence reveals that the location at which Trk receptors are activated may play a pivotal role in determining the precise downstream significance of Trk activation. For example, BDNF-induced increase in primary dendrites was recently demonstrated to involve TrkB activation in the lipid rafts [13]. In addition, retrograde transport of activated Trk receptors as signaling endosomes is emerging as a key regulator of neuronal survival [29]. Since we examined changes in TrkB downstream signaling cascades only in total lysates, it remains possible that Cdk5 activity may specifically affect TrkB signaling only at certain subcellular/plasma membrane compartments.

Secondly, overexpression of CA Cdc42 restored BDNF-induced dendritic growth in *cdk5* ^−/−^ neurons and in neurons overexpressing TrkB M1 (Figure 6), suggesting that maintenance of Cdc42 activation was sufficient to overcome the lack of BDNF-stimulated dendritic growth when Cdk5-mediated TrkB phosphorylation was absent. It thus appears that Cdk5 may impair BDNF-induced Cdc42 activation by affecting activation of the Rho GTPase. On the other hand, it should also be noted that overexpression of both the DN and CA forms of Cdc42 had a negligible effect on the basal number of primary dendrites in both *cdk5* ^+/+^ and *cdk5* ^−/−^ neurons (Figure 6). Our observation is in agreement with an earlier study demonstrating that overexpression of DN or CA Cdc42 had no effect on the number of primary dendrites in chick spinal neurons [30]. In addition, it is consistent with the observation that modulation of Cdk5 activity or overexpression of TrkB M1 affected only BDNF-induced dendritic growth, without affecting the basal number of dendrites. Nonetheless, the inability of CA Cdc42 to mimic BDNF in the induction of primary dendrites suggests that activation of Cdc42 per se was insufficient to trigger dendritic growth in the absence of BDNF, and that additional, BDNF-dependent event(s) are required for the induction of dendritic growth by BDNF. Although the precise pathways implicated remain to be identified, it is tempting, in light of the emerging importance of lipid rafts in the activation of Rho GTPase, to speculate that BDNF may be required to stimulate translocation of activated Cdc42 to lipid rafts. In support of this hypothesis, it was observed that activation of Rac1 depends on the translocation of the activated Rho GTPase to lipid rafts [25,26]. In addition, in the absence of cholesterol, CA Rac1 failed to translocate to plasma membrane in fibroblasts [25]. More importantly, depletion of cholesterol similarly abolished BDNF-induced increase in primary dendrites in hippocampal neurons [13]. These observations collectively suggest that the inability of CA Cdc42 to increase dendritic growth in the absence of BDNF treatment may be related to the lack of CA Cdc42 translocation to lipid rafts, which may potentially be induced by BDNF treatment. A thorough investigation of the importance of lipid rafts in Cdc42 activation and primary dendrite induction by BDNF will shed light on the mechanisms by which BDNF-triggered dendritic growth is regulated.

Given the near absence of Ser478-phosphorylated TrkB in *cdk5* ^−/−^ brain, we believe that Cdk5 functions as the predominant kinase for this phosphorylation in vivo. Nonetheless, it was interesting to note that prior to BDNF stimulation, a basal level of Ser478-phosphorylated TrkB was detected in cortical neurons that was not inhibited by pretreatment with the Cdk5 inhibitor Ros. This may suggest that other serine kinases are present to phosphorylate TrkB at Ser478 in the absence of BDNF stimulation. Nonetheless, given the marked inhibition of BDNF-stimulated increase in TrkB phosphorylation by Ros, we believe that Cdk5 is essential for the BDNF-dependent component of TrkB phosphorylation at Ser478.

Given the abundant expression of Cdk5 and TrkB in neurons throughout development, and their respective concentration at the synapse, it would be interesting to examine if Cdk5 activity is also involved in other downstream functions of TrkB signaling, such as the regulation of neuronal survival and synaptic plasticity. Preliminary findings from our laboratory reveal that Cdk5 activity is also required for BDNF-stimulated neuronal survival in cortical neurons (unpublished data). In addition, the juxtamembrane region of Trk receptors has been associated with the regulation of Trk receptor internalization [31] and degradation [32]. Further investigation of whether this phosphorylation also affects the internalization and degradation of the receptor would provide further insights into the biological significance of this phosphorylation. In addition, since Cdk5 was observed to associate with TrkA without phosphorylating the receptor, further delineation of the consequences of this interaction would be essential for thoroughly understanding the crosstalk between Trk receptors and Cdk5. A preliminary study revealed that, similar to TrkB, TrkA phosphorylates Cdk5 at Tyr15 (unpublished data). The differential interaction of TrkA and TrkB with Cdk5, together with the differential localization of TrkA and TrkB in different neuronal populations, may provide a novel mechanism by which Cdk5 can regulate the signaling of different neuronal populations. In conclusion, our findings have provided evidence for a regulatory role of Cdk5 in Trk-induced dendritic growth, and lend support for an emerging role of Cdk5 as a regulator of RTK signaling. Given the importance of neurotrophin/Trk signaling in almost all aspects of neuronal development and function, our findings will likely have far-reaching implications for further elucidating the signaling mechanisms involved in the regulation of neuronal survival, synapse formation, and synaptic plasticity.

## Materials and Methods

### Antibodies, DNA constructs, and siRNAs.

The antibodies against Trk (C-14), Cdk5 (DC-17), p35, and Shc were purchased from Santa Cruz Biotechnology (http://www.scbt.com). The antibodies against TrkB and SH2B were from BD Biosciences (http://www.bdbiosciences.com). The polyclonal antibodies recognizing phospho-TrkA (Tyr490), p44/42 mitogen-activated protein kinase (Erk1/2), phospho-p44/42 mitogen-activated protein kinase, AKT, phospho-AKT (Ser473), CREB, and phospho-Ser133 CREB were obtained from Cell Signaling Technology (http://www.cellsignal.com). Antibodies specific for actin and β-tubulin type III were from Sigma-Aldrich (http://www.sigmaaldrich.com). Antibody against the p-Ser478 of TrkB was raised by synthetic peptide (CISNDDDSApSPLHHIS; Bio-Synthesis, http://www.biosyn.com) and purified using AminoLink Kit (Pierce, http://www.piercenet.com).

Expression vectors of p35, Cdk5, and DN Cdk5 were prepared as previously described [3]. Flag-tagged and GST-tagged Cdk5 were generated by PCR, and subcloned into the mammalian expression vectors pcDNA3 (Invitrogen, http://www.invitrogen.com) and pGEX-6P-1 (Amersham Biosciences, http://www5.amershambiosciences.com), respectively. HA-tagged and GST-tagged Rac1, Cdc42, and RhoA constructs were gifts from Yung-Hou Wong (Hong Kong University of Science and Technology, Hong Kong). The expression vectors of TrkA, TrkB, and TrkC were constructed as described [33]. Three TrkB mutants lacking the potential Cdk5 phosphorylation sites were constructed by mutating Ser478 (TrkB M1), Thr489 (TrkB M2), or both Ser478 and Thr489 (TrkB DM) to alanine using the overlapping PCR technique, followed by subcloning into pcDNA3. GST-TrkB-Juxta construct was generated by PCR and subcloned into pGEX-6P-1. Protein purification was performed according to the manufacturer's protocol.

Stealth RNAi molecules for Cdk5 were prepared as previously described [34]. The sequences used were: Cdk5 siRNA, CCUCCGGGAGAUCUGUCUACUCAAA; and control siRNA (Cdk5), CCUAGGGCUAGCUGUUCAUCCCAAA.

### Animals, primary cultures, and transfection.

Cdk5 and p35 knockout mice were kindly provided by A. B. Kulkarni (National Institutes of Health, Bethesda, Maryland) and T. Curran (St. Jude Children's Research Hospital, Memphis, Tennessee), and L. H. Tsai (Harvard Medical School, Boston, Massachusetts), respectively. Mice from different stages were collected and genotyped as described [7,35].

Rat cortical and hippocampal neuron cultures were prepared as previously described [33,34]. Subsequent to digestion with 0.25% trypsin in Hank's Balanced Salt Solution without Ca^2+^ and Mg^2+^ at 37 °C for 5 min, the reaction was stopped by 2.5% heat-inactivated horse serum. The dissociated neurons were seeded in culture dishes coated with 10 μg/ml poly-D-lysine. Two hours later the medium was replaced by neurobasal medium supplemented with 2 mM L-glutamine and 2% B27 supplement.

Selective Cdk5 inhibitor Ros (Calbiochem, http://www.merckbiosciences.com/html/CBC/home.html) was used to inhibit Cdk5 activity in primary neuron cultures. Primary cultures at 3 d in vitro (DIV3) were treated with or without BDNF (50 ng/ml) in the presence of Ros (10 or 25 μM) or DMSO for 3 d before harvesting or fixation.

For transfection of primary cultures, cortical and hippocampal neurons were seeded on coverslips in 12-well dishes at a cell density of 2 × 10^5^ per coverslip. Neurons were transfected using calcium phosphate precipitation at DIV3. Twenty-four hours after transfection, the cultures were treated with BDNF for 3 d.

Primary hippocampal neuron cultures on coverslips in 12-well dishes were seeded at a cell density of 5 × 10^4^ per coverslip for siRNA transfection. Cultures were transfected at DIV3 with Lipofectamine 2000 transfection reagent following the manufacturer's protocols (Invitrogen). The transfected cells were incubated at 37 °C with 5% CO_2_ for 24 h before treatment, and were then treated with BDNF for 3 d.

### Cell cultures and transfection.

COS7 cells and HEK293T cells were obtained from American Type Culture Collection (http://www.atcc.org). Both cells were maintained in DMEM supplemented with 10% heat-inactivated fetal bovine serum, penicillin (50 units/ml), and streptomycin (100 μg/ml) at 37 °C with 5% CO_2_. COS7 cells and HEK293T cells were transfected using Lipofectamine Plus transfection reagents following the supplier's instructions (Invitrogen). The cells were treated and harvested 24 h after transfection.

### Protein extraction, immunoprecipitation, in vitro pull-down assay, and Western blot analysis.

Cells were lysed at 4 °C for 30 min in lysis buffer (RIPA: 1× PBS, 1% NP40, 0.1% SDS, and 0.5% sodium deoxycholate) with various protease inhibitors (1 mM phenylmethylsulfonyl fluoride [PMSF], 1 mM sodium orthovanadate [NaOV], 2 μg/ml antipain, 10 μg/ml leupeptin, 30 nM okadaic acid, 5 mM benzamidine, and 10 μg/ml aprotinin). Brain tissues were homogenized in lysis buffer (0.5% NP-40, 20 mM Tris-HCl, 150 mM NaCl, 1 mM EDTA, 1 mM EGTA, and 1 mM NaF [pH 7.5]) supplemented with various protease inhibitors (1 mM PMSF, 1 mM NaOV, 2 μg/ml antipain, 10 μg/ml leupeptin, 30 nM okadaic acid, 5 mM benzamidine, and 10 μg/ml aprotinin). Proteins were resolved by SDS-PAGE and subsequently electro-transferred onto a nitrocellulose membrane. Immmunoblots were probed with the desired primary antibodies at 4 °C overnight. After washing with TBS-T, the corresponding HRP-conjugated secondary antibody was added and incubated for 2 h at room temperature. Proteins were then visualized using enhanced chemiluminescence Western blotting detection reagents with reference to the supplier's instructions (Amersham Biosciences).

For immunoprecipitation, 1–2 mg of protein lysates was incubated with 1 μg of the corresponding antibody at 4 °C overnight with rotation. Forty microliters of protein G Sepharose (Amersham Biosciences) pre-washed with 1× PBS was added and rotated at 4 °C for 1 h. After intense washing with the lysis buffer, the immunoprecipitated protein and its associated proteins were analyzed by SDS-PAGE and Western blotting.

Flag-tagged protein was overexpressed in COS7 cells and the cell lysate was obtained as described above. The cell lysate obtained was incubated with anti-Flag M2 affinity gel (Sigma-Aldrich) at 4 °C overnight with rotation. The Flag-tagged protein was pulled down by the affinity gel, and the affinity gel was washed twice with lysis buffer (0.5% NP-40, 20 mM Tris-HCl, 150 mM NaCl, 1 mM EDTA, 1 mM EGTA, and 1 mM NaF [pH 7.5]) with various protease inhibitors (1 mM PMSF, 1 mM NaOV, 2 μg/ml antipain, 10 μg/ml leupeptin, 30 nM okadaic acid, 5 mM benzamidine, and 10 μg/ml aprotinin). One to two milligrams of proteins prepared from brain tissues was incubated with the affinity gel, and the Flag-tagged protein pulled down by the affinity gel for 1 h. The affinity gel was washed twice with lysis buffer supplemented with protease inhibitors. The proteins pulled down by the Flag-tagged protein were subjected to Western blot analysis.

### In vitro kinase assay.

Recombinant Cdk5/p35 and Cdk5/p25 were kindly provided by Shin-Ichi Hisanaga (Tokyo Metropolitan University, Tokyo). TrkA, TrkB, and TrkC were immunoprecipitated from transfected HEK293T cells, and used as substrates for reconstituted Cdk5/p35 or Cdk5/p25 in the in vitro kinase assay. The kinase assay was performed at 30 °C for 30 min in kinase buffer containing 100 μM [γ-^32^P] ATP as described [36]. To examine if TrkB phosphorylated Cdk5, recombinant TrkB kinase domain (Upstate Biotechnology, http://www.upstate.com) was incubated with GST-Cdk5 for 30 min at 30 °C, with or without Trk inhibitor K252a pretreatment (100 nM) for 10 min, in the presence of 100 μM [γ-^32^P] ATP or cold ATP. To examine if BDNF stimulated Cdk5 activity, primary cortical neurons were treated with BDNF with or without 30 min of K252a pretreatment (100 nM). The immunoprecipitated Cdk5/p35 complexes from the lysates were washed three times with lysis buffer and twice with kinase buffer. The in vitro kinase reaction was performed at 30 °C for 30 min with kinase buffer containing 100 μM histone H1 peptide and 100 μM [γ-^32^P] ATP as described [37]. The phosphorylated proteins were resolved by SDS-PAGE. After the gel was dried, the phosphorylated proteins were visualized by autoradiography. For TrkB-mediated Cdk5 phosphorylation, the phosphorylated protein was resolved by SDS-PAGE, and blotted with phospho-Cdk2 (Tyr15; Santa Cruz Biotechnology) or phosphotyrosine antibody (4G10; Upstate Biotechnology).

### GTPase activity assay.

GTPase activity was measured as described [38]. Briefly, cultured cortical neurons at DIV7 were pretreated with DMSO or Ros for 30 min, followed by treatment with BDNF for another 5 min. Cells were lysed at 4 °C and incubated with Pak1-PBD agarose with constant rocking at 4 °C for 1 h. The proteins bound to the beads were washed three times with lysis buffer at 4 °C, eluted in SDS sample buffer, and analyzed for bound Cdc42 by Western blotting using monoclonal antibody against Cdc42 (Upstate Biotechnology). GTPase activity was quantified by densitometry analysis of the blots.

### Immunohistochemical analysis.

Following fixation in 4% paraformaldehyde and 5% sucrose in PBS with Ca^2+^ and Mg^2+^ for 30 min, the cells were washed three times with PBS, and were blocked with 1% bovine serum albumin and 10% goat serum for 20 min. The cells were then incubated with the corresponding primary antibody (1:150–500) at 4 °C overnight, and were subsequently washed with PBS three times. Following incubation with FITC or rhodamine conjugated secondary antibody (1:1,000) for 1 h at room temperature, the cells were washed again, stained with DAPI, and mounted with coverslips and MOWIOL (Calbiochem). Mounted cells were visualized under fluorescent microscope (Leica, http://www.leica.com).

### Statistical analysis.

All data were expressed as mean ± standard deviation. Statistical significance was determined by one-way analysis of variance followed by Bonferroni's post hoc test with 95% confidence. A *p*-value of smaller than 0.05 was considered as statistically significant.

## Abbreviations

BDNF: brain-derived neurotrophic factor
CA: constitutively active
Cdk5: cyclin-dependent kinase 5
DIV[number]: [number] days in vitro
DN: dominant negative
GEF: guanine nucleotide exchange factor
NaOV: sodium orthovanadate
NGF: nerve growth factor
NT-[number]: neurotrophin-[number]
p-Ser478: phospho-Ser478
P7: postnatal day 7
PMSF: phenylmethylsulfonyl fluoride
Ros: roscovitine
RTK: receptor tyrosine kinase
siRNA: short interferring RNA
WT: wild-type
